# Health care experiences of female sex workers: a qualitative study

**DOI:** 10.1093/fampra/cmaf048

**Published:** 2025-06-19

**Authors:** Özlem Kızıltaş, İzzet Fidancı, Hilal Aksoy, Duygu Ayhan Başer

**Affiliations:** Seyhan Health Directorate, Adana, Turkey; Department of Family Medicine, Faculty of Medicine, Hacettepe University, Ankara, Turkey; Department of Family Medicine, Faculty of Medicine, Hacettepe University, Ankara, Turkey; Department of Family Medicine, Faculty of Medicine, Hacettepe University, Ankara, Turkey

**Keywords:** sex workers, access to health services, family practice, sexual activity, qualitative research, patient satisfaction

## Abstract

**Background:**

Sex workers face significant barriers to accessing health services, including stigma, economic constraints, and safety concerns. In Turkey, this group is often subjected to discrimination and prejudiced approaches when accessing sexual health services, which reduces the uptake of health services. This study aims to analyze the health care experiences of sex workers in depth.

**Methods:**

The study conducted semi-structured in-depth interviews with 16 women working as sex workers in Adana brothel in July–October 2024, and the data were analyzed through thematic analysis. Participants were selected through purposive sampling to ensure socio-demographic diversity.

**Results:**

Four main themes were identified through the analysis: Barriers to Access and Use of Health Services, Health Service Experiences and Satisfaction, Information and Awareness, and Emotional Situations. Participants indicated that they often preferred private health facilities due to difficulties in accessing public health services and long waiting times, but that these preferences were limited by cost.

**Conclusions:**

Improving the quality of public health services and reducing costs may improve public health by encouraging this group to use health services.

**Key messages**

- Sex workers face systemic and emotional barriers in public health services.
- Long waits and rigid systems drive preference for private health care access.
- Health needs include chronic, mental, and aesthetic care—not just sexual health.
- Trust, confidentiality, and non-judgmental care are critical for engagement.
- Inclusive, patient-centered primary care is vital to improve health engagement.

## Background

Sex work shows great differences in terms of both legality and social perception throughout the world. This situation causes sex workers to face various difficulties in accessing health services. Especially stigma, discrimination, legal restrictions, economic reasons, and prejudices towards health services reduce the rate of application to health services by female sex workers and negatively affect health outcomes [1, 2].

Sex workers are at high risk of physical and sexual health problems. The risk of contracting diseases, such as sexually transmitted infections, HIV, and hepatitis is higher for this group. Despite these risks, sex workers have very low rates of access to health services. Fear of stigma, legal restrictions, and prejudiced attitudes of health service providers are the most important of these difficulties [1].

Sex work is legal in Turkey, but it is known that sex workers face several barriers to accessing health services. One of the main barriers to accessing health services is the way public health institutions work, working hours, and economic barriers. For this reason, female sex workers generally prefer private health services, but this may cause a financial burden [2].

This study aims to understand the access and usage habits of female sex workers to health services and the barriers they face in this process. The findings to be obtained are expected to contribute to a better understanding of the health needs of female sex workers and to the development of more effective health policies in this area.

## Methods

Ethical approval was granted by the Adana City Training and Research Hospital Scientific Research Evaluation Commission (Meeting number: 2, Date: 26.06.2024, Decision number: 46) and the Adana Provincial Health Directorate granted approval (Number: E-96172664-050.06-99 229097044, Date: 09.08.2024, Decision number: 6) prior to the commencement of the qualitative study.

This study adhered to the EQUATOR (Enhancing the QUAlity and Transparency Of health Research) Network, specifically the Consolidated Criteria for Reporting Qualitative Research (COREQ) checklist, to ensure transparency and rigor are upheld in this research. Participants were purposively sampled to capture variations in socio-demographic characteristics, such as age, educational background, and professional experiences. Semi-structured interviews were designed and conducted according to COREQ recommendations, ensuring confidentiality and comfort for participants during data collection. Ethical approval was provided by the institutional review boards, with written informed consent obtained from all participants prior to the interviews. Thematic data analysis was performed, which was further enhanced in terms of credibility and reliability by iterative processes of coding and categorization, using COREQ criteria. The inclusion of direct participant quotes further supports the authenticity of the findings. In line with these guidelines, this study also contributes to health care literature with robust, transparent qualitative evidence [3–6] (Supplementary Material).

The research team was led by senior physicians with advanced training in qualitative research methods and public health. All interviews were conducted by a female physician specializing in sexual and reproductive health, maintaining a professional demeanor, expressing appropriate empathy when interacting with participants. The interviewer had never met the participants before the interviews and thus held no prejudice toward them at the time of the data collection. The interviewer was non-judgmental and supportive to ensure participants felt safe talking about their experiences. Reflexivity was actively practiced throughout the research process by acknowledging and addressing any preconceived beliefs held by the research team. These were implemented to ensure transparency and rigor as proposed in COREQ guidelines [4, 6].

This qualitative study aims to explore in depth how female sex workers working in the Adana brothel access health services, their patterns of service use, and the challenges they face throughout this process. Qualitative research methods provide an appropriate approach to understanding the experiences and emotions of the participants, and semi-structured in-depth interview techniques were used in this study.

The study group consisted of women aged 18 and above who live in Adana and are engaged in sex work. Interviews were conducted with participants who had been examined at the dermatology and venereal diseases clinic, after they had been informed about the study and had given their consent. A total of 16 participants were interviewed between July and October 2024, and the sample size was determined when thematic saturation was reached.

### Data collection method

Data were collected using a semi-structured interview form. The interview questions were prepared to understand the participants’ access to health services, the barriers they encountered, the extent to which they were satisfied with the services, and the emotional and psychological conditions they experienced in this process. The interviews were conducted in environments where the participants felt comfortable and each interview lasted 25–30 minutes on average. The interviews were recorded with a voice recorder and then transcribed by considering their facial expressions and emotional states during the conversation.

### Data availability statement

The data underlying the findings of this study are available from the corresponding author upon reasonable request. Due to the sensitive nature and agreements on confidentiality with participants regarding the information provided, no raw data have been shared.

### Data analyses

The collected data were analyzed using thematic analysis. Firstly, the recurring codes in the interviews were identified, and then these codes were grouped into main themes according to their similarities. Four main themes emerged in this study: Barriers to Access and Use of Health Services, Health Service Experiences and Satisfaction, Information and Awareness, and Emotional Situations.

## Results

A purposive sampling technique was used in the selection of the participants. The participants were selected among individuals who are engaged in sex work and have experience in accessing sexual health services. Our study was completed by interviewing 16 participants. The mean age of the participants was 43.1 ± 9.6 (min = 28; max = 65) and the mean number of children was 0.9 ± 0.6 (min = 0; max = 2). Socio-demographic data, age at first sexual intercourse, and contraceptive method are shown in Table 1. Thirteen (81.3%) of the participants were smokers, 7 (43.7%) were alcohol users, and no drug users were found. Six (50%) of the participants had chronic diseases and these participants were continuously taking medication. The diagnosed conditions were asthma, fibromyalgia, anxiety disorder, depression, epilepsy, hypertension, hypercholesterolemia, SLE (systemic lupus erythematosus), and hypothyroidism.

**Table 1.** Sociodemographic and sexuality information of the participants.

Number of participants	Age	Marital status	Number of children	Educational status	Health insurance	Duration of employment (years)	Age at first sexual intercourse	Contraceptive method
*Participant 1*	40	Single	0	Primary school	Yes	14	15	Condom
*Participant 2*	49	Divorced	1	Secondary school	Yes	20	15	Condom
*Participant 3*	54	Divorced	1	Primary school	Yes	29	18	None (menopause)
*Participant 4*	34	Single	0	High school	Yes	7	17	Condom
*Participant 5*	52	Divorced	2	Primary school	Yes	18	16	OCs
*Participant 6*	65	Spouse deceased	1	High school	Yes	23	16	None (menopause)
*Participant 7*	36	Divorced	1	Secondary school	Yes	4	18	Condom
*Participant 8*	28	Single	1	Primary school	Yes	3	16	Condom
*Participant 9*	46	Single	0	High school	Yes	12	17	OCs
*Participant 10*	46	Single	1	University	Yes	10	17	Condom
*Participant 11*	31	Single	1	Secondary school	Yes	8	19	Norplant
*Participant 12*	51	Divorced	1	Primary school	Yes	10	14	Condom
*Participant 13*	37	Single	1	High school	Yes	12	15	OCs
*Participant 14*	41	Single	1	University	Yes	17	16	Condom
*Participant 15*	41	Single	2	Secondary school	Yes	9	19	OCs
*Participant 16*	38	Single	0	High school	Yes	17	16	IUD

OCs: Oral contraceptives, IUD: Intrauterine device.

In this study, sex workers’ access to and use of health services and the difficulties they face in these processes are grouped under four main themes: Barriers to Access and Use of Health Services, Health Service Experiences and Satisfaction, Information and Awareness, and Emotional Situations (Table 2). In each theme, the findings are deepened by including the direct statements of the participants.

**Table 2.** Themes and sub-themes.

Main theme	Sub-themes
***Barriers to Access and Use of Health Services***	Work-related request
	Attending a family doctor
	The institution where the health service is received
	Reason for choosing a health institution
	How to choose a health institution
	Difficulties in accessing health services
	Problems related to health
	Financial factors
***Health Service Experiences and Satisfaction***	Access to health services
	Reason for the health service they want to receive
	The health service they want to receive
	Healthy behavior
	Attitude towards work
	How to choose a health institution
	Evaluation of previous health care
	Evaluation of the screening health service received
	Satisfaction
	Evaluation of previous health care
***Information and Awareness***	Awareness
	Information
	Disease knowledge
	Dissatisfaction
***Emotional Situations***	Satisfaction
	Sadness
	Stress
	Anxiety

### Barriers to access and use of health services

Participants stated that they faced various obstacles in accessing health services. Financial difficulties, professional demands, and difficulties in accessing health services attracted attention.

One of the participants stated that state hospitals and family health centers are an important factor in the reasons why they are preferred because they do not burden patients economically:

‘I mean, I would prefer it in any case. I mean, it is both financially good and we meet good doctors.’ (Participant 3)

Although 6 of the participants said that they did not use family health centers, 60% of them said that they preferred family health centers for simple medical interventions, upper respiratory tract infections, vaccination services, and women’s health check-ups aged 15–49. In terms of preference for family health centers, 9 participants said that they went to family health centers especially for prescription:

‘And I go once a week, for example, to use suppositories, and since I tell the family doctor that I am doing this job, I go to them to prescribe suppositories or I don’t know, prescribe a cream.’ (Participant 11)

‘Generally, because I have a chronic illness, I go to health centers to get my medication prescribed when I have a medical report.’ (Participant 5)

While 75% of the participants answered the question of the institution where they receive health services as private hospitals, they expressed the reason for turning into private hospitals as follows:

‘In public hospitals, they do not attach importance to human health, whereas in private hospitals they provide much more attentive care.’ (Participant 1)

‘They show more interest, they show a lot of interest, for example, special, okay, money is expensive, but they show a lot of interest. It really happens for him. The state hospital is not interested at all.’ (Participant 12)

‘I think that good physicians are no longer left in the state, I think that good physicians no longer work in state hospitals. I think they all resigned and moved to private hospitals.’ (Participant 14)

‘I believe that public hospitals were finished years ago. All the good doctors were taken by private companies, and the better ones have already gone abroad.’ (Participant 4)

The 41-year-old university graduate participant, who stated that she could not access health services, stated that the reason for this was that doctors could not spare enough time for patients due to working conditions:

‘Maybe because of the diseases and disorders I have experienced, I can say that there is nothing wrong with me, so I think physicians do not have time. Each of them has to look after 50, 60 patients a day.’ (Participant 14)

Another factor in access to and use of health services is economic conditions, and the participants stated that economic conditions were effective in their preference for private health institutions, while others stated that the hours of access to this service were incompatible with their work schedules.

### Health service experiences and satisfaction status

Most of the participants stated that there was no inability to access or receive health services, but that working hours were an obstacle to access to health services:

‘Appointments do not fit my working system. I cannot get service at the time I want; I must use private services.’ (Participant 10)

For this reason, the same participant stated that she received therapy with a psychiatrist from a different province over the internet.

‘I got help from him at work, via Zoom.’ (Participant 10)

One participant expressed the process of receiving health services from the family physician as follows:

‘It is difficult to go to the family doctor, I must be there at 8 a.m., but I cannot go because I have work. I wish there were more flexible hours.’ (Participant 10)

Participants emphasized that their healthcare behavior was often driven by their needs and professional demands. Some stated that they specifically seek sexual health products and psychological support:

‘Condoms are very costly, I could buy them from the health center, but now I have to buy them myself.’ (Participant 12)

Another 37-year-old participant emphasized the need for psychological support and said:

‘I need to talk, to commiserate. I need more psychological support, not medication.’ (Participant 13)

In addition, most of the participants stated that they wanted to look aesthetically good due to their jobs and expressed their demands related to this:

‘I would like aesthetics in state hospitals. They can do breast aesthetics. They can do hip aesthetics.’ (Participant 11)

‘Yes, after that I like aesthetics. But I care more about body aesthetics than facial aesthetics. Because for us, showing off is very important. Image is very important.’ (Participant 1)

While the participants expressed their satisfaction with private hospitals while receiving healthcare services, they emphasized the negative experiences they had in public hospitals. A 28-year-old primary school graduate participant said the following about the interest in private hospitals:

‘They take much better care in private hospitals, I pay a ton of money, but at least I am greeted with a smiling face.’ and ‘There is nothing in public hospitals, no hygiene, no interest. Health service should not be provided like this.’ (Participant 8)

In this study conducted in Adana brothel, individuals drew attention to the lack of health services and expressed their demands:

‘In Istanbul, they used to give us special lessons on condoms, for example, when we went there, on TV, doctors used to tell us to use condoms.’ (Participant 1)

When asked to evaluate the screening examinations conducted in the outpatient clinic room in the working areas of sex worker women as a skin and venereal diseases unit, one of the participants stated that she worked in a brothel because of these screening examinations:

‘I have no discomfort at all. And I would not choose a brothel anyway, this examination, blood, good attention.’ (Participant 15)

All participants gave positive feedback about the health screenings:

‘I find it very good for our future in terms of health, for our children and for not harming some other people to be examined 2 days a week, to have our blood taken in 3 months, to have our x-rays taken in 6 months.’ (Participant 3)

‘I am glad that we are already being examined 2 times a month, 2 times a week really, and at least it is a precaution. For me, it is a very good thing for people for protection.’ (Participant 13)

### Information and awareness

Participants expressed that they lacked information and awareness in the process of utilizing health services. Especially regarding sexually transmitted diseases and preventive health services, a participant expressed this situation as follows:

‘I used to be able to buy condoms and lubricant gel from health centers, but now they don’t give them to me. I must buy them with my money.’ (Participant 12)

Another participant drew attention to the demand for gynecological support and regular check-ups and stated the following:

‘Gynecological support is very important, regular check-ups should be done, but not enough attention is paid to this in public hospitals.’ (Participant 14)

One participant explained that after the pandemic she paid more attention to her health:

‘I mean, even if it’s normal flu outside, I pay attention. I stay away from those people. I don’t kiss them or anything. We became more aware after the pandemic.’ (Participant 7)

The occupational demands of the participants directly affected health service utilization. Some participants stated that they turned to private health services because the demand for sexual health products was not met. A participant expressed this as follows:

‘Condoms and gel are costly; the state should provide them free of charge. We need these while doing our job.’ (Participant 10)

### Emotional situations

The emotional reactions of the participants during the process of accessing health services were frequently associated with dissatisfaction, surprise, and sadness. One participant expressed her dissatisfaction with the lack of interest in public hospitals with the following words:

‘The doctor never looked at me, he just prescribed medicine and sent me away. Without saying anything, without questioning. This made me very sad.’ (Participant 8)

Another participant expressed her surprise with the following words:

‘You go to the state hospital, but they treat you as if you are not there, I am really surprised by this situation.’ (Participant 13)

The women who participated in the study stated that they had negative emotional experiences while using health services and that this situation caused them to move away from health services. One participant described the lack of interest in state hospitals as follows:

‘They made me wait in the state hospital, the doctor was not interested at all and responded to me by singing a song. Health service should not be like this.’ (Participant 8)

Such experiences led to dissatisfaction and anxiety among the participants.

## Discussion

In this study, the processes related to female sex workers’ access to and use of health services (working hours, appointments, waiting times, economic conditions, and psychological conditions, which are the main difficulties they face) have been comprehensively addressed. The results show that work-related demands, preference for family medicine, methods of choosing health institutions, and financial barriers play a crucial role in the participants’ access to health services.

Easily accessible health services help reduce occupational stigma and discrimination, increasing sex workers’ use of these services [7]. Public, safe, and confidential health services enable them to meet their health needs more effectively. High-quality, timely, and patient-centered primary care is well-suited to addressing their unmet needs [8]. As Kim et al. emphasize, women-centered, non-judgmental spaces specifically designed for sex workers facilitate access to care [9]. Flexible service hours are also crucial, especially for night workers, ensuring access to both preventive and curative services at any time [7, 10]. In Hong Kong, some sex workers preferred non-governmental organization-run clinics that offered emotional support and free sexual health supplies, citing prejudice in public health services as a barrier [11]. Moreover, negative experiences in primary care may push sex workers toward emergency services, disrupting continuity of care [12].

The findings of this study underscore the strategic importance of primary health care services—particularly family medicine practices—in facilitating the integration of sex workers into the health system. While a portion of participants reported utilizing family health centers primarily for prescription refills or minor medical interventions, fears of stigma, limited communication, and inflexible appointment structures significantly hindered broader engagement with these services. Yet, primary care, with its person-centered, continuous, and comprehensive service model, constitutes the most appropriate framework for delivering inclusive and equitable care to vulnerable populations. Harris et al. demonstrated that a structurally inclusive and non-discriminatory primary care system was positively associated with increased and sustained health service utilization among sex workers [8]. In this context, ensuring that family medicine services are accessible and professionally equipped in areas, such as sexual health, mental health, and the prevention of infectious diseases will not only strengthen the health of sex workers but also contribute to the protection of public health and reinforce primary health care services.

Economic barriers are also among the key factors limiting access to health services. Although public health institutions are more cost-effective, participants reported difficulties in accessing private health services due to their high costs. The literature indicates that economic constraints particularly hinder access to sexual health services and increase the risk of infectious diseases [13, 14]. Therefore, maintaining low costs in public health services, offering certain services free of charge, and implementing flexible service hours can significantly improve access [7, 10].

In this study, participants frequently expressed a need for mental health services, which they associated directly with their occupational experiences. A notable finding was their high awareness of this need. Treloar et al. found that while sex workers often seek mental health support, service providers tend to view sex work as the root cause of all issues, failing to recognize the individual’s broader identity [15]. This perspective leads to feelings of exclusion in mental health settings. Interestingly, some participants in our study reported that openly stating their profession during therapy helped build a more effective therapeutic relationship. However, there remains a need for more comprehensive research into the mental health needs of sex workers, particularly in the context of their working conditions.

Social exclusion, stigmatization, discrimination, and violence negatively impact the general and sexual health of sex workers, creating significant barriers to accessing health services [16]. Additionally, issues such as lack of contraception, unwanted pregnancies, miscarriages, and repeated abortions further compromise their reproductive health [17]. Platt et al. also stated that being defined as a sex worker decreases the rate of application to health services by such vulnerable groups and this situation increases sexual health risks [14, 18].

It should be emphasized that sex workers require a holistic medical approach not only in sexual and mental health but also in managing general health conditions such as chronic diseases [11]. Providing sensitivity training to all healthcare professionals can enhance both the acceptability and effectiveness of services [12]. Culturally safe healthcare environments have been shown to increase service utilization, making health services more accessible and acceptable, thereby encouraging broader and more consistent use [19–21].

There is underinvestment in both services and research, particularly for non-street sex workers. However, a range of interventions are likely to be effective. Services should be developed and delivered in co-operation with sex workers. Interventions that focus on education and empowerment or that have multiple components are likely to be effective, and in some cases, an outreach or direct engagement component may be beneficial [22]

Although drug use is commonly observed in other studies conducted on sex workers, there are also studies in which nicotine use is common supporting the results of this study [23, 24].

This study has certain limitations. Since the participants were selected from a specific region and socioeconomic background, the findings may not be generalizable. Additionally, due to the stigma surrounding sex work, some participants may have refrained from sharing sensitive experiences. This may have resulted in some aspects of health care access remaining unexplored. Future research should involve more diverse populations and adopt a longer-term and more comprehensive approach to better inform policy development in this area.

## Conclusion

To ensure equal access to health care among female sex workers, inclusive, culturally safe, and non-discriminatory service environments must be established with support from trained health care providers. Elimination of economic barriers and offering flexible, free sexual and reproductive health services will significantly enhance the utilization of services. Integration of sex workers into the health system using stigma-free, women-centered, and comprehensive primary care models will not only improve individual health but also enhance public health activities.

## Supplementary Material

cmaf048_suppl_Supplementary_Material_1

cmaf048_suppl_Supplementary_Material_2
